# Modifying response times in the Simon task with transcranial random noise stimulation

**DOI:** 10.1038/s41598-017-15604-1

**Published:** 2017-11-15

**Authors:** James Robert McIntosh, Carsten Mehring

**Affiliations:** 1 Faculty of Biology, University of Freiburg, Hansastr. 9A, 79104 Freiburg, Germany; 2 Bernstein Center Freiburg, University of Freiburg, Hansastr. 9A, 79104 Freiburg, Germany; 3 Department of Bioengineering, Imperial College of Science, Technology and Medicine, South Kensington, London, SW7 2AZ UK

## Abstract

Perceptual decisions pervade our every-day lives, and can align or conflict with inbuilt biases. We investigated these conflicting biases by applying transcranial random noise stimulation (tRNS) while subjects took part in a visual Simon task - a paradigm where irrelevant spatial cues influence the response times of subjects to relevant colour cues. We found that tRNS reduces the response time of subjects independent of the congruence between spatial and colour cues, but dependent on the baseline response time, both between subjects and across conditions within subjects. We consider the reduction in response time to be non-specific to the Simon task, and cast our interpretations in terms of drift-diffusion models, which have been previously used as mechanistic explanations for decision-making processes. However, there have been few extensions of the drift-diffusion model to the Simon effect, and so we first elaborate on this interpretation, and further extend it by incorporating the potential action of tRNS.

## Introduction

In common perceptual decision making tasks one of two responses has to be made, for example on the basis of the direction of motion of coherent random dots^1,2^. In such a case, evidence for a response in one direction is thought to count against evidence for a response in the opposite direction. One potential extension of such a paradigm is to consider the development of a decision when the response depends, or is thought to depend on two sources of information which are in direct conflict. We design an experiment using the “Simon effect”, coined by Hedge *et al*.^3^ in which conflict arises when a subject must react to relevant information regarding the response, which is however presented in a location inconsistent with that response. The conflict generally leads to an increase in response time (RT). This bias due to conflict was first measured by Simon *et al*.^4^, and has since been repeated with many variations, including with single handed responses in left or right directions^5^; responses to tones as opposed to words^6^; and with visual as opposed to auditory stimuli^7–9^. What is common in all these cases, is that in some manner, a source of information which is clearly irrelevant to the task is interfering with a relevant source.

Here, we use the visual Simon effect, where the conflict in this case appears to be between the intended response based on the colour of the cue, and an automatic response based on the side to which the cue is presented. Because in this version of the Simon effect the visual stimuli and the response are lateralised, we decided to apply transcranial random noise stimulation (tRNS) across the two hemispheres with the hypothesis that we may be able to influence conflict resolution, and take advantage of long cortical-cortical axonal projections between the hemispheres as potentially sensitive targets.

Transcranial random noise stimulation has been previously used to modify motor excitability in humans, for example, Terney *et al*.^10^ showed that motor-evoked potentials (MEP) generated by transcranial magnetic stimulation (TMS) had increased amplitude when compared to sham following the application of tRNS, and suggested that the influence was dominated by higher frequencies. Fertonani *et al*.^11^ showed that tRNS with frequencies between 100 and 640 Hz improved performance accuracy in an orientation discrimination task, confirming the general effectiveness of the technique to modify behaviour. Recent work^12^ showed that application of tRNS with an electrode positioned over the occipital cortex initially enhanced performance in a visual detection task, but as amplitude was increased, the visual detection worsened, consistent with concepts of stochastic resonance.

Computational models to capture the Simon effect have been previously put forward^13,14^. However generally such models do not capture the full RT distributions in correct and incorrect trials, and are difficult to directly integrate with models of the function of tRNS. Recently, attempts have been made to extend the drift-diffusion model framework^15–17^ (DDM) to conflict tasks^18–20^. Particularly for the Simon effect, this is not immediately possible due to a reversed relationship between mean RT and standard deviation across conditions when compared to the DDM. The diffusion model for conflict tasks^20^ (DMC) is able to cater to this, at the cost of being difficult to interpret. Here we propose a simpler DDM based model which fully captures our RT distributional data, and we then augment this with an additional noisy input to cater for changes in RT due to stimulation.

We found that the influence of tRNS in our experimental condition modifies RT, however not in a way that is specific to the congruent or incongruent conditions of the Simon task. More generally we found that the influence on RT across conditions is dependent on the baseline RT, consistent with the addition of noise in evidence accumulator based interpretations of the Simon effect.

## Methods

### Participants

24 subjects (7 female) aged between 20 and 40 participated. The experimental procedures were approved by the ethics committee of the University of Freiburg. All participants gave informed consent after being briefed on transcranial current stimulation and filling out an exclusion questionnaire. All methods and procedures were performed in accordance with the relevant guidelines and regulations. Two subjects were determined to be left handed on the Edinburgh scale and were therefore excluded from our analysis.

### Task

**Figure 1:**
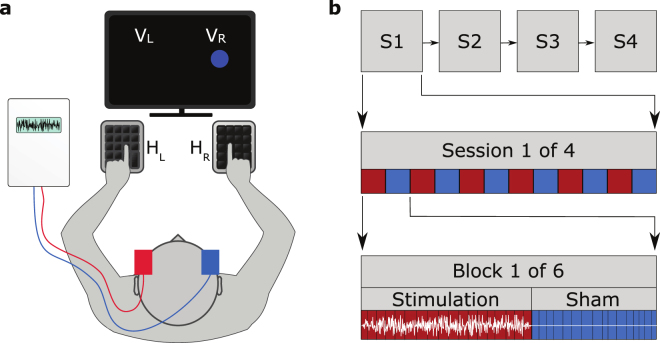
Experimental setup and design for applying tRNS while a subject performs the Simon task. (**a**) The subject must press a key with the left hand (*H*_*L*_) in response to a blue visual cue, and with the right hand (*H*_*R*_) in response to a yellow visual cue, irrespective of the cue presentation side (visual hemifield, *V*_*L*_ or *V*_*R*_). Application of tRNS is via sponge electrodes positioned at FT7 and FT8 on the EEG 10–20 system. (**b**) Experiment is composed of four identical sessions (S1-S4), each session is broken up into six blocks which are in turn composed of 16 tRNS trials (red), 16 sham trials (blue) and two transition trials (not shown).

Our paradigm was based on a modification of the Simon effect task^4^. Subjects sat in front of a monitor while fixating a cross placed in its centre (see Fig. 1a). They were required to wait for time intervals chosen pseudorandomly between 1.8 s and 3.2 s in 0.2 s steps, the choice was made so that each interval was selected four times within one block and equally often between stimulation and sham conditions. They were then shown a coloured cue in either their left or their right visual hemifield for 50 ms (three frames on our 60 Hz monitor). The subject’s task was to respond as quickly to the cue colour as possible, pressing a key with their left hand (*H*_*L*_) for one colour, and with their right hand (*H*_*R*_) for the other, while ignoring the physical location of the cue (left *V*_*L*_, or right *V*_*R*_).

Subjects were asked to respond as quickly as possible and avoid making mistakes. RT with reference to visual cue location and hand response were recorded. The experiment was divided into four sessions (see Fig. 1b), at the end of each session subjects were allowed to take a break. A single session was divided into six blocks, each composed of 34 trials. Additionally, we randomised whether the experiment began with sham or stimulation, and randomly assigned subjects to one of two difficulty levels with respect to a maximum constraint of 0.6 s or 0.7 s for RT. When subjects failed to respond in time, or made an incorrect key-press a tone was played. All experiments were conducted using the Psychophysics Toolbox extension^21–23^ for MATLAB.

### Transcranial random noise stimulation

Out of the 34 trials in one block, we applied tRNS stimulation for 18 consecutive trials (see Fig. 1). The first and last trial of tRNS within each block were used to ramp up and ramp down the tRNS amplitude respectively, and were discarded from the analysis. During the remaining 16 trials in each block we applied no stimulation.

Stimulation was delivered to the head via saline soaked sponge electrodes (7 cm × 5 cm) positioned at FT7 and FT8 in the EEG 10–20 system. The tRNS was generated by triggering a DC Stimulator Plus (neuroConn GmbH, Ilmenau, Germany) with a National Instruments DAQ (National Instruments, USA). The stimulator generates the waveform such that for every sample point (1280 Hz) a value is drawn from a Gaussian distribution where there is a 99% probability of being within a fixed range of −500 *μ*A to 500 *μ*A, with the absolute value having a hard ceiling of 600 *μ*A. The resultant distribution is to all practical intents and purposes the same as a Gaussian distribution and consequently has a flat spectrum.

### Analysis

#### Pre-processing

Key-presses that were performed less than 0.2 s after cue onset were removed as they were deemed to occur only if the subject initiated the movement prior to the stimulus being presented. We also did not analyse key-presses where the subject failed to answer within the required RT, and pooled data across our two RT difficulty levels. Unless stated otherwise, we also removed key-presses where subjects made an incorrect response.

#### Stimulation influence on response time and accuracy

In order to determine the influence of stimulation, as well as the influence of visual hemifield stimulus laterality and response hand, on the RT averaged for each subject and condition, we fitted a linear mixed-effects model as shown in equation (1).

1 $$R{T}_{is}={\beta }_{0s}+{\beta }_{1s}V+{\beta }_{2s}H+{\beta }_{3s}V\cdot H+{\beta }_{4}S+{\beta }_{5}V\cdot S+{\beta }_{6}H\cdot S+{\beta }_{7}V\cdot H\cdot S$$

The model was designed to fit the mean RT over observations (*i*) taking into account all interactions incorporating the visual hemifield (*V*), response hand (*H*) and stimulation (*S*) factors. We also included random effects for subject-specific constants and slopes (enumerated via the variable *s*) on the interaction between visual hemifield and response hand, such that the mixed-effects parameters in equation (1) take the form *β*_*js*_ = *β*_*j*0_ + *b*_*js*_ where ${b}_{js}\sim N(\mathrm{0,}\,{\sigma }_{j}^{2})$. We chose this random effects structure to account for variability in the asymmetries in RT across subjects, for example due to the degree of visual hemifield or hand dominance while keeping the model complexity as low as possible.

To assess the influence of stimulation on accuracy we extended the linear mixed-effects model described in equation (1) by incorporating a logistic link function in order to describe the probability of making a correct choice as shown in equation (2).

2 $$log(\frac{p}{1-p})={\beta }_{0s}+{\beta }_{1s}V+{\beta }_{2s}H+{\beta }_{3s}V\cdot H+{\beta }_{4}S+{\beta }_{5}V\cdot S+{\beta }_{6}H\cdot S+{\beta }_{7}V\cdot H\cdot S$$

#### Stimulation influence dependence on baseline response time

We were interested in the baseline RT in the sham condition as a potential covariate to explain the change in RT due to stimulation. To assess the relationship between the average RT across conditions for each subject, and the average RT change across conditions induced by stimulation, we used a robust linear fit (using Tukey’s bisquare loss function). To assess this, we performed a t-test to determine whether the slope of the fit was significantly different from zero. As an additional control to address a potential dependence of ΔRT on RT due to a statistical effect of regression towards the mean, we also repeated the slope fit by randomising the assignment of sham and stimulation to each condition (10^4^ iterations). We then counted the proportion of iterations less than the slope estimated from the non-shuffled data. This is equivalent to the p-value for rejecting the null hypothesis that our measured slope is not more negative than a slope that would be generated from the random assignment of stimulation and sham.

Finally, to examine the same covariate within subjects we constructed a linear mixed-effects model (equation 3) with factors RT, visual hemifield, response hand and their interactions in order to explain the change in RT between the sham and stimulation condition (ΔRT). We included random effects of offset and slope on the RT which were dependent on subject. We note that the choice of the random effects structure only has minor effects on the result of this statistical test, and we decided to not include visual hemifield and response hand as factors to avoid over-parametrising the model.

3 $${\rm{\Delta }}R{T}_{is}={\beta }_{0s}+{\beta }_{1s}RT+{\beta }_{2}H+{\beta }_{3}V+{\beta }_{4}V\cdot H+{\beta }_{5}V\cdot RT+{\beta }_{6}H\cdot RT+{\beta }_{7}V\cdot H\cdot RT.$$

#### Simon effect

In order to address whether stimulation impacted the measured Simon effect, we once again resorted to using a linear mixed-effects model with random effects of constant and slope dependent on subject. In this case, the factors are stimulus-response congruence and stimulation.

4 $${\rm{\Delta }}R{T}_{is}^{S{E}_{V}}={\beta }_{0s}+{\beta }_{1s}S{E}_{V}+{\beta }_{2}S+{\beta }_{3}S{E}_{V}\cdot S$$

5 $${\rm{\Delta }}R{T}_{is}^{S{E}_{H}}={\beta }_{0s}+{\beta }_{1s}S{E}_{H}+{\beta }_{2}S+{\beta }_{3}S{E}_{H}\cdot S$$

We fitted one linear model for each type of Simon effect, that is with the dependent variable *stimulus* Simon effect (${\rm{\Delta }}R{T}_{is}^{S{E}_{V}}$, equation 4), and with the dependent variable *response* Simon effect (${\rm{\Delta }}R{T}_{is}^{S{E}_{H}}$, equation 5). The *stimulus* Simon effect on the left side, is defined as the RT in the *V*_*L*_ *H*_*R*_ condition minus the RT in *V*_*L*_ *H*_*L*_ condition (*V*_*L*_ *H*_*R*_ − *V*_*L*_ *H*_*L*_), while the *stimulus* Simon effect on the right side is defined as the RT difference *V*_*R*_ *H*_*L*_ − *V*_*R*_ *H*_*R*_. Similarly, the left *response* Simon effect is defined as the RT difference *V*_*R*_ *H*_*L*_ − *V*_*L*_ *H*_*L*_, while the right *response* Simon effect is defined as the RT difference *V*_*L*_ *H*_*R*_ − *V*_*R*_ *H*_*R*_.

### Behavioural model

#### Design

We decided to characterise the Simon effect in terms of a modified DDM, in order to test how introducing noise (tRNS) into such a system would influence RT. We were interested in finding out whether enhanced noise, which in a DDM can lead to earlier threshold crossings would have an influence dependent on certain parameter settings in our model. In particular, we were interested in whether parameters which generate fast simulated average RT, are less influenced than those which generate longer simulated average RT.

**Figure 2:**
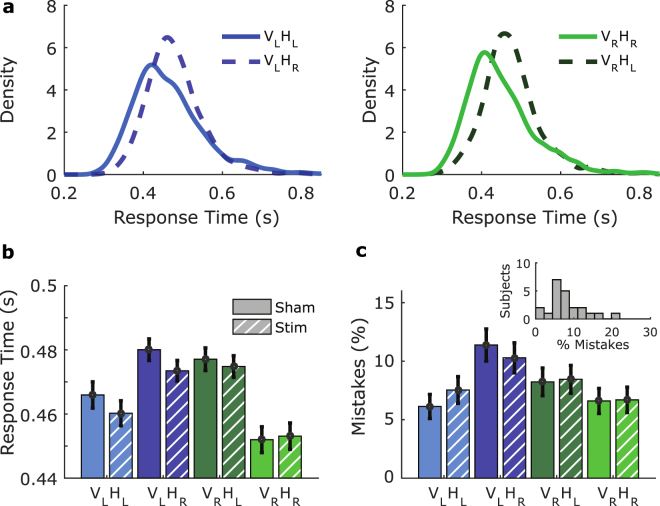
Simon effect, and the basic influence of stimulation. (**a**) Simon effect RT distributions show density estimates of individual subject normalised RT, multiplied by average RT across subjects. Congruent conditions, *V*_*L*_ *H*_*L*_ and *V*_*R*_ *H*_*R*_ (solid lines) are left shifted and more skewed than the incongruent conditions *V*_*L*_ *H*_*R*_ and *V*_*R*_ *H*_*L*_ (dashed lines). Normalisation of RT is done by dividing by the average for each subject, density estimation of RT distributions were generated by convolution with a Gaussian kernel with a fixed standard deviation of 15 ms. (**b**) Mean of individual subject normalised RT, multiplied by grand mean RT across subjects for different experimental conditions. A general trend of reduction in RT is visible across conditions with tRNS, and is clearest in the *V*_*L*_ *H*_*R*_ condition. The Simon effect is visible in that the central incongruent conditions have longer RT than the congruent conditions. Error bars represent 95% CI. (**c**) A change in accuracy due to tRNS is difficult to discern, although mistakes appeared to be increased in the incongruent condition when compared to the congruent condition, independent of stimulation as would be expected. Error bars represent 95% CI. Inset: Histogram of mistakes for individual subjects. On average (median) subjects made mistakes in 6% of trials during the task.

Our model is based on the DDM^15–17^ and follows in the steps of previous work^18–20^ in applying a DDM framework to the Simon task. The DDM predicts linear (or near linear) increases in standard deviation with increasing mean^24,25^. However, this relationship is violated between congruent and incongruent conditions in the Simon effect^19,26–28^ (see Fig. 2a). Specifically, in the incongruent condition with respect to the congruent condition in the Simon effect, the mean increases while the standard deviation of the RT distribution decreases. At high RT the Simon effect has even been reported to invert because of this reduction in standard deviation^27,29^.

In designing a DDM based model for the Simon effect, we initially attempted to capture a shift in attention towards the irrelevant stimulus by introduction of a bias in the starting decision-variable. This notion stems from summarising the key property of a brief and automatic activation towards the stimulus source (a component of several Simon effect models and theories^6,20,26,30^), without a strong regard for its temporal profile. A simple bias in starting decision-variable however is not sufficient to capture the intricacies of the Simon Effect as it introduces a stronger Simon effect influence at longer RT, something which has been reported to be inconsistent with Simon effect data^26^, and is indeed inconsistent with our findings. In the DMC^20^, this problem is dealt with via the introduction of a drift with a specific temporal profile and while this fully captures the RT distributions it may be difficult to fully falsify due to its flexibility.

As a solution to a simple bias in decision-variable being insufficient to capture the RT distribution, while attempting to avoid over specifying the temporal evolution of the drift, we were inspired by the shrinking-spotlight (SSP) model^18^. This model has been used to effectively describe the RT distributions in the Eriksen flanker task, although it failed to capture the intricacies of the Simon effect task in its original form^19^. In the SSP, distracter stimuli influence the development of a decision-variable because at the start of a trial they are captured by attention. However, as the trial progresses attention shrinks to focus on the relevant stimulus effectively boosting it and suppressing the irrelevant stimuli. Here, we consider an alternative which is that the presence of conflict recruits additional attention as a trial progresses, hence enhancing the effective drift. Intuitively, our proposed model works because a starting bias in the decision-variable acts as a primary source of conflict reducing the mean RT in the conflict trials, however the introduction of boosted attention in these same trials then reduces the standard deviation. One potentially undesirable property of our model is that the presence of conflict, which is not known a priori is a factor that enables the recruitment of attention. However, we do not consider this to be a fundamental problem as the impact of this factor is zero at the start of the trial so that similar mechanisms that govern the development of the decision-variable for choice could also govern the development of a decision-variable for conflict, which would additionally allow us to remove the direct temporal dependence.

Our model can be summarised as follows:

6 $${\rm{\Delta }}x=I\cdot H(1+\frac{1-V\cdot H}{2}b\cdot t){\rm{\Delta }}t+c{\xi }_{1}\sqrt{{\rm{\Delta }}t}$$

7 $${x}_{0}={x}_{0}^{bias}\cdot V$$

8 $${t}_{d}={t}_{d}^{base}+{\xi }_{2}{c}_{td}$$

Equation (6) describes the dynamics of the decision-variable (*x*). A change over a single time step Δ*t* (set to be 1 ms) is governed by a drift term *I*, a constant term which is multiplied by the factor *H* which is set to take a value of −1 or +1 dependent on the colour of the visual cue, and consequently the correct response. Similarly the term *V* is set to take a value of −1 or +1 dependent on location of the visual cue. Consequently, the term (1 − *V* · *H*)/2 is 0 during a congruent trial and 1 during an incongruent trial, signalling the recruitment of additional attention with the passage of time *t*, implemented by a constant slope *b*. The use of a constant for the base drift term *I* makes the assumption that the brief, 50 ms visual stimulus persists in a local memory and continues to feed the evidence accumulator as time passes^31,32^. The dynamics of the decision-variable are also governed by a noise term *c* fixed at 0.1 which acts to scale a Gaussian noise process ${\xi }_{1}\sim N\mathrm{(0,}\,\mathrm{1)}$.

Equation (7) describes the starting value of the decision-variable *x*_0_ at time *t*_*d*_, which is dependent on a constant bias term ${x}_{0}^{bias}\cdot V$, where the sign depends on the visual hemifield to which the cue is displayed due to the *V* term.

Finally, equation (8) describes the non-decision time *t*_*d*_ to allow for a period of processing time after stimulus presentation but before evidence integration, as well as for a certain amount of movement time. This parameter is composed of *t*_*d*_^*base*^, and also has an associated variability drawn from a uniform distribution ${\xi }_{2}\sim U(-\frac{1}{2},\frac{1}{2})$, and scaled by a term *c*_*td*_.

As is typical in the DDM framework, a decision is considered to have been made when the decision-variable crosses a threshold. Our convention is to use a crossing of +*x*_*th*_ for a rightwards movement, or −*x*_*th*_ for a leftwards movement.

To summarise, the model distinguished between experimental conditions (excluding tRNS for now), via the terms *V* and *H*. Six parameters are left free to be fitted (*I*, *x*_*th*_, *t*_*d*_^*base*^, *c*_*td*_, *b*, and ${x}_{0}^{bias}$) of which two parameters (*b*, ${x}_{0}^{bias}$) govern the difference in RT distributions between the different experimental conditions.

#### Model fits

We generated bins with edges defined by the average 10^*th*^, 30^*th*^, 50^*th*^, 70^*th*^ and 90^*th*^ percentiles of the data across subjects for the data corresponding to correct key presses for each condition. We then computed the RT distributions for each condition generated by 25 × 10^3^ iterations of our model, and calculated the corresponding relative proportion of correct RT falling into each bin. We repeated the same procedure for the incorrect key presses, however in this case we only attempted to use a single bin edge (50^*th*^ percentile) due to the relatively small amount of available data. A cost function computed from the sum of the *χ* ^2^ statistic across the bins^33^, and averaged across conditions was then minimised by using MATLAB’s implementation of the generalised pattern search algorithm^34^. In an attempt to reduce the influence of local minima we re-initiated the minimisation procedure with randomised initial values. Once we had generated a fit for the population data, we also attempted to fit individual subject data with the same model. In this case, we initiated our model parameters to match the parameters found from the population data.

Additionally, after fitting the model to the sham data, we incorporated tRNS by augmenting equation (6) with the term *Aξ*_*tRNS*_ $\sqrt{(\Delta t)}$ to form equation (9):

9 $${\rm{\Delta }}x=I\cdot H(1+\frac{1-V\cdot H}{2}b\cdot t){\rm{\Delta }}t+(c{\xi }_{1}+A{\xi }_{tRNS})\sqrt{{\rm{\Delta }}t}$$

where *A* is a scaling term corresponding to the strength of stimulation, and *ξ*_*tRNS*_ represents a noise term like *ξ*_1_, although it is independent of it. The size of the stimulation strength parameter *A* used in our simulations was determined by choosing the value that yielded the lowest *χ* ^2^ statistic on the full data set (sham and stimulation), after having fixed all other parameters with model fits on the sham data.

To investigate the influence of stimulation across individual subjects as fitted by our DDM based model we determined whether a linear relationship held between the change in RT induced by stimulation (ΔRT), and the baseline sham RT. In order to determine which parameters may be of interest in modifying the influence of RT on our subjects, we also used linear fits to determine which parameters in our model varied systematically across the RT of our subjects. Parameters that across subjects systematically varied with RT (averaged across conditions) were then individually investigated by evaluating their impact on the influence of stimulation using the same intensity value as determined for our pooled data.

#### Visual drift-asymmetry - extended model

By design, the model presented thus far is symmetrical in that it makes identical predictions for both congruent conditions, and separately, identical predictions for both incongruent conditions. While this may be suitable as a first approximation to the Simon effect, it is clear that this is in fact not the case^35^. There are several options for introducing asymmetries into the model, for example, by adding condition dependent non-decision times, thresholds, or drifts. Furthermore, the condition dependent parameters may depend on visual hemifield, response hand, or the interaction of the two. We chose to investigate the drift-asymmetry model (which we refer to as the extended model), as it provides an intuitive explanation for our within subject changes in RT, which is a natural extension of our simpler symmetrical model. We note however, that this choice will have to be verified independently in future work, and is currently beyond the scope of this study. Mathematically, the drift asymmetry is introduced by assigning *I* from equation (6), *I* = *I*_*a*_ + *fH* + *gV*. During model fits on the pooled data, the parameter *f* was found to be near zero, and we consequently simply fixed it to zero resulting in our model’s free parameter *I*, being replaced with *I*_*a*_ and *g*.

#### Sensitivity to stimulation

In order to determine whether within subject differences in RT across conditions have an impact on the influence of stimulation, we define the following three measures:

10 $${{\rm{Sensitivity}}}_{{\rm{1}}}=\frac{{\rm{1}}}{{\rm{2}}}(\frac{{{\rm{\Delta }}\mathrm{RT}}_{{{\rm{V}}}_{{\rm{L}}}{{\rm{H}}}_{{\rm{L}}}}-{{\rm{\Delta }}\mathrm{RT}}_{{{\rm{V}}}_{{\rm{R}}}{{\rm{H}}}_{{\rm{R}}}}}{{{\rm{RT}}}_{{{\rm{V}}}_{{\rm{L}}}{{\rm{H}}}_{{\rm{L}}}}-{{\rm{RT}}}_{{{\rm{V}}}_{{\rm{R}}}{{\rm{H}}}_{{\rm{R}}}}})+\frac{{\rm{1}}}{{\rm{2}}}(\frac{{{\rm{\Delta }}\mathrm{RT}}_{{{\rm{V}}}_{{\rm{L}}}{{\rm{H}}}_{{\rm{R}}}}-{{\rm{\Delta }}\mathrm{RT}}_{{{\rm{V}}}_{{\rm{R}}}{{\rm{H}}}_{{\rm{L}}}}}{{{\rm{RT}}}_{{{\rm{V}}}_{{\rm{L}}}{{\rm{H}}}_{{\rm{R}}}}-{{\rm{RT}}}_{{{\rm{V}}}_{{\rm{R}}}{{\rm{H}}}_{{\rm{L}}}}})$$

11 $${{\rm{Sensitivity}}}_{{\rm{2}}}=\frac{{\rm{1}}}{{\rm{2}}}(\frac{{{\rm{\Delta }}\mathrm{RT}}_{{{\rm{V}}}_{{\rm{L}}}{{\rm{H}}}_{{\rm{L}}}}-{{\rm{\Delta }}\mathrm{RT}}_{{{\rm{V}}}_{{\rm{L}}}{{\rm{H}}}_{{\rm{R}}}}}{{{\rm{RT}}}_{{{\rm{V}}}_{{\rm{L}}}{{\rm{H}}}_{{\rm{L}}}}-{{\rm{RT}}}_{{{\rm{V}}}_{{\rm{L}}}{{\rm{H}}}_{{\rm{R}}}}})+\frac{{\rm{1}}}{{\rm{2}}}(\frac{{{\rm{\Delta }}\mathrm{RT}}_{{{\rm{V}}}_{{\rm{R}}}{{\rm{H}}}_{{\rm{R}}}}-{{\rm{\Delta }}\mathrm{RT}}_{{{\rm{V}}}_{{\rm{R}}}{{\rm{H}}}_{{\rm{L}}}}}{{{\rm{RT}}}_{{{\rm{V}}}_{{\rm{R}}}{{\rm{H}}}_{{\rm{R}}}}-{{\rm{RT}}}_{{{\rm{V}}}_{{\rm{R}}}{{\rm{H}}}_{{\rm{L}}}}})$$

12 $${{\rm{Sensitivity}}}_{{\rm{3}}}=\frac{{\rm{1}}}{{\rm{2}}}(\frac{{{\rm{\Delta }}\mathrm{RT}}_{{{\rm{V}}}_{{\rm{L}}}{{\rm{H}}}_{{\rm{L}}}}-{{\rm{\Delta }}\mathrm{RT}}_{{{\rm{V}}}_{{\rm{R}}}{{\rm{H}}}_{{\rm{L}}}}}{{{\rm{RT}}}_{{{\rm{V}}}_{{\rm{L}}}{{\rm{H}}}_{{\rm{L}}}}-{{\rm{RT}}}_{{{\rm{V}}}_{{\rm{R}}}{{\rm{H}}}_{{\rm{L}}}}})+\frac{{\rm{1}}}{{\rm{2}}}(\frac{{{\rm{\Delta }}\mathrm{RT}}_{{{\rm{V}}}_{{\rm{R}}}{{\rm{H}}}_{{\rm{R}}}}-{{\rm{\Delta }}\mathrm{RT}}_{{{\rm{V}}}_{{\rm{L}}}{{\rm{H}}}_{{\rm{R}}}}}{{{\rm{RT}}}_{{{\rm{V}}}_{{\rm{R}}}{{\rm{H}}}_{{\rm{R}}}}-{{\rm{RT}}}_{{{\rm{V}}}_{{\rm{L}}}{{\rm{H}}}_{{\rm{R}}}}})$$

where RT and ΔRT are calculated as the mean across trials for individual conditions. The sensitivity metrics are calculated for each subject individually for both RT data and extended model fits unless stated otherwise.

#### Model selection

In order to provide a rigorous motivation for our model selection we considered how the average *χ* ^2^ value would change for alternative model configurations. We defined a base model to be a simple DDM, including variability in the non-decision time:

13 $${\rm{\Delta }}x=I\cdot H{\rm{\Delta }}t+c{\xi }_{1}\sqrt{{\rm{\Delta }}t}$$

14 $${x}_{0}=0$$

15 $${t}_{d}={t}_{d}^{base}+{\xi }_{2}{c}_{td}$$

This base model takes the same structure as our model from equations (6–8), with the same parameter definitions, although only includes parameters that are common in perceptual decision making models, and omits parameters that are specific to the Simon effect and its asymmetries. Starting from this base model, we then generated a model for every combination of parameters that we proposed to include. These are the two additional parameters introduced for our simple Simon effect model (${x}_{0}^{bias}$, *b*), and two additional parameters that we introduced for our extended model (*f* response hand asymmetry, *g* visual hemifield asymmetry). We evaluated the *χ* ^2^ statistic of each model (*n* = 16) and for every subject individually (*n* = 22) using 8-fold cross-validation. Fitting was done as previously described, using the model parameters from the across subject model fit as starting values. Additionally, we repeated the procedure 2 more times with random starting values (by adding a random value drawn from a Gaussian distribution scaled to be 20% of the original values) in an attempt to reduce the probability of our results being due to local minima. We performed Wilcoxon signed-rank tests on the averaged cross-validated *χ* ^2^ statistic across subjects in order to determine if chosen progressively more complex models showed clear improvements.

### Data Availability

All experimental data generated or analysed during this study are included in this published article (and its Supplementary Information files).

## Results

### tRNS influences response time in the Simon effect

Figure 2a shows density estimates of RT distributions which demonstrate the typical Simon effect. That is, RT in the incongruent conditions (*V*_*L*_ *H*_*R*_ and *V*_*R*_ *H*_*L*_), are significantly longer than in the congruent conditions (*V*_*L*_ *H*_*L*_ and *V*_*R*_ *H*_*R*_).

To determine the influence of tRNS, we fitted a linear mixed-effects model with the following factors: stimulation (tRNS, Sham), response hand (*H*_*L*_, *H*_*R*_), and visual hemifield (*V*_*L*_, *V*_*R*_), as well as with random effects dependent on the average subject RT for correct trials. We found a significant main influence of visual hemifield (*p* = 0.0398), response hand (*p* = 0.0099) and stimulation (*p* = 0.0156). We also found significant effects of interactions between visual field and hand (*p* = 6 × 10^−4^), but not of any other interaction (see Table 1).

**Table 1:** Mixed-effects linear model statistics for fixed effects on RT with factors of visual hemifield, response hand, and stimulation, as well as using random effects for subject-specific constants and slopes.

Factor	Estimate	SE	p
Visual hemifield (V)	0.012	0.005	0.0398
Response hand (H)	0.014	0.005	0.0099
Stimulation (S)	−0.007	0.003	0.0156
V · H	−0.041	0.010	6 × 10^−4^
V · S	0.005	0.004	0.228
H · S	0.000	0.004	0.998
V · H · S	0.005	0.006	0.375

Model is fitted to within subject and condition averages (n = 176).

Figure 2b shows RT averages across subjects, after individual subject normalisation, and then multiplied by the subject grand mean RT. Again, this figure demonstrates the Simon effect, including a typically seen stronger influence in the right Simon effect when compared to the left Simon effect for both visual hemifield, and response hand (compare dark bars to light bars). A general influence of stimulation is also visible when examining the mean RT, particularly for the *V*_*L*_ *H*_*R*_ condition.

### Influence dependence on response time

**Figure 3:**
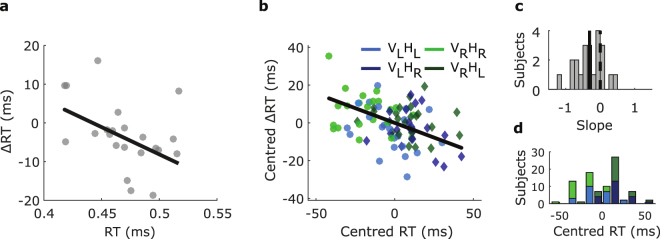
The influence of stimulation on RT depends on the baseline RT. (**a**) Negative ΔRT corresponds to shorter RT during stimulation, each point represents a single subject where their RT, as well as the change in RT due to stimulation has been averaged across conditions. Change in RT between sham and stimulation appears to be negatively correlated to the baseline RT (black line: slope = −0.141 ± 0.057, *p* = 0.023). (**b**) Within subjects, baseline RT also has a large impact on the influence of stimulation. Each point corresponds to a single condition for each subject. A slope is fitted to explain ΔRT in terms of RT for each subject, and the black line shows the average across all subjects. (**c**) The distribution of slopes fitted in (**b**) across subjects. The vertical black line corresponds to the slope of (**b**) and the dashed line is centred at zero. The distribution is significantly asymmetrical (Wilcoxon signed-rank test, *p* = 0.0015, mean = −0.31). (**d**) Histogram of centred RT, showing the relatively longer RT of the conflict conditions.

The noticeable differences of the influence of stimulation visible in Fig. 2b, particularly between *V*_*L*_ *H*_*R*_ and *V*_*R*_ *H*_*R*_ led us to hypothesise about mechanisms that could yield subtle changes across conditions. Specifically, we considered whether longer sham RT (which we refer to as baseline RT) might lead to larger changes in RT induced by stimulation (which we will refer to as ΔRT). In order to test this, we initially examined the ΔRT dependence on the baseline RT averaged across conditions between subjects, as shown in Fig. 3a. Here, we found a dependence of ΔRT on RT (slope = −0.141 ± 0.057, *p* = 0.023, Fig. 3a), suggesting that this may be a viable and more general interpretation of our results. We were concerned that a general negative slope in the dependence of ΔRT on RT may occur naturally as a product of regression towards the mean, i.e. that points with extreme RT in the baseline (sham) are statistically likely to be coupled with less extreme points in stimulation. To address this, we repeated the slope fits of Fig. 3a, randomising assignments of sham and stimulation to each condition (see methods). We found that our real slope was significantly less than the mean of the randomised slope distribution (*p* = 0.021, proportion of iterations less than real slope), confirming that our influence of RT on ΔRT is not caused by a statistical effect of regression towards the mean.

In order to examine the relation between RT and ΔRT within subjects, taking the visual hemifield and response hand conditions into account, we fitted a linear mixed-effects model with the dependent variable as the change in RT between sham and stimulation. The factors in this case were the baseline RT in the sham condition, visual hemifield, response hand and their interactions. We also included a random effects term for the offset and slope dependent on subject. We found this test to yield a significant dependence of ΔRT on RT (slope = −0.24 ± 0.08, *p* = 0.003), signifying that the variation in RT within subjects is an important factor for the susceptibility of the subject’s RT to stimulation. We also found no significant effect of any other factor or interaction (see Table 2). In order to visualise the impact that stimulation has dependent on the baseline RT within subjects, in Fig. 3b we plot ΔRT against RT for each subject and condition, after subtracting the average across conditions respectively for each subject. A line is then fitted to the centred ΔRT across the four conditions for each subject. The black line in Fig. 3b, corresponding to the vertical black line in Fig. 3c has a slope set to be the average of these fits, with the full distribution shown in Fig. 3c (Wilcoxon signed-rank test, *p* = 0.0015). This once again shows that on average, for each subject ΔRT is more negative for larger RT.

**Table 2:** Mixed-effects linear model statistics for fixed effects on ΔRT, with factors visual hemifield, response hand, and RT, as well as using random effects for subject-specific constants and slopes.

Factor	Estimate	SE	p
Response time (RT)	−0.24	0.08	0.003
Visual hemifield (V)	−0.05	0.04	0.271
Response hand (H)	−0.02	0.05	0.687
V · RT	0.12	0.09	0.201
H · RT	0.05	0.10	0.623
V · H	−0.03	0.07	0.692
V · H · RT	0.06	0.15	0.696

Model is fitted to within subject and condition averages (n = 88).

### Influence on accuracy

Figure 2c shows the percentage of mistakes in each condition averaged across subjects. Clearly, incongruent trials produce more mistakes, although it is difficult to discern a systematic influence of stimulation on the pooled data. Subjects generally performed to a very high standard, and made very few mistakes as is shown in the inset of Fig. 2c, with the median percentage of incorrect responses being approximately 6%.

In order to investigate more rigorously whether stimulation impacts accuracy, we used a generalised linear mixed-effects model to account for the average accuracy of subjects in each condition. The model was built with factors of visual hemifield, response hand, stimulation as well as their interactions. Once again, we also included a random effects term for subject-specific constants and slopes. The summary statistics are shown in Table 3. The strongest influence on the accuracy is dependent on the response hand (*p* = 0.003). We also detected a significant influence of the interaction between response hand and stimulation (*p* = 0.028), however we are cautious to interpret this due to the additional complexity introduced in the model by the non-linear component, together with the relatively small range of accuracies across subjects which is close to the maximum possible accuracy (100%).

**Table 3:** Statistics for generalised linear mixed-effects model to account for accuracy with factors of visual hemifield, response hand, and stimulation and their interactions, as well as using random effects for subject-specific constants and slopes.

Factor	Estimate	SE	p
Visual hemifield (V)	−0.105	0.260	0.688
Response hand (H)	−0.645	0.213	0.003
Stimulation (S)	−0.228	0.126	0.071
V · H	0.642	0.462	0.167
V · S	0.190	0.171	0.269
H · S	0.359	0.162	0.028
V · H · S	−0.394	0.236	0.098

Model is fitted to within subject and condition averages (n = 176).

### tRNS does not detectably influence the Simon effect

The Simon effect is composed by taking the difference between the incongruent condition and the congruent condition, however this can be considered from two points of view: the *stimulus* Simon effect where we fix the stimulus laterality, and the *response* Simon effect where we fix the required response hand^35^.

If we consider the *stimulus* Simon effect (equation 4) for the left side: *V*_*L*_ *H*_*R*_ − *V*_*L*_ *H*_*L*_, RT from both conditions are reduced, making the difference due to tRNS largely unchanged, while for the right side: *V*_*R*_ *H*_*L*_ − *V*_*R*_ *H*_*R*_, stimulation does not appear to change RT. Fitting a linear mixed-effects model (n = 88), with visual hemifield as a factor, and subjects as random effects, on the mean differences in RT we find no significant effect of stimulation. Specifically, we find an influence of visual field (slope = −0.041 ± 0.010, *p* = 6.0 × 10^−5^), suggesting an asymmetry in the *stimulus* Simon effect, and no influence of stimulation (slope = 0.000 ± 0.004, *p* = 1.00), or its interaction with visual field (slope = 0.005 ± 0.005, *p* = 0.32).

If we consider the *response* Simon effect (equation 5) for the left side: *V*_*R*_ *H*_*L*_ − *V*_*L*_ *H*_*L*_, then stimulation acts to increase this value (as the congruent RT moves further from the incongruent RT), while for the right side: *V*_*L*_ *H*_*R*_ − *V*_*R*_ *H*_*R*_, stimulation acts to decrease this difference (as the incongruent RT moves closer to the congruent RT). A linear mixed-effects model fit was repeated as above, however now with response hand as a factor instead of stimulus laterality. In this case, we find an influence of response hand (slope = −0.041 ± 0.010, *p* = 5.7 × 10^−5^), but no influence of stimulation (slope = 0.005 ± 0.003, *p* = 0.16), and no influence in the interaction term (slope = 0.005 ± 0.005, *p* = 0.30).

It appears then that our application of tRNS does not have a systematic influence on the Simon effect, and that we should therefore interpret our changes in RT either in a condition dependent way, or dependent on the baseline RT.

### Model captures the Simon effect and predicts dependence on response time

**Figure 4:**
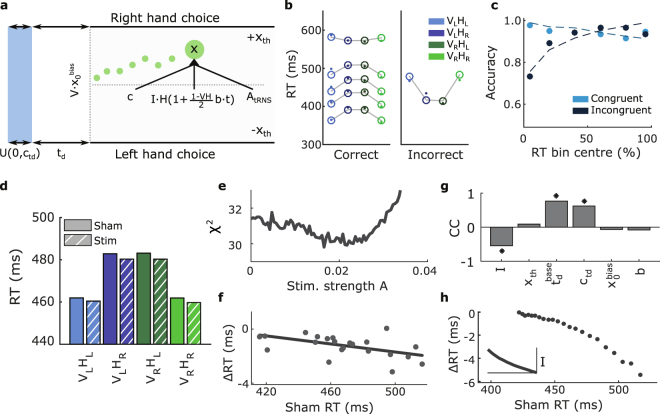
Model fit and influence of stimulation. (**a**) Structure of our model as described in equations (6–8). (**b**) Model fit across conditions for both correct (left: 10^*th*^, 30^*th*^, 50^*th*^, 70^*th*^ and 90^*th*^ percentiles), and incorrect (right: 50^*th*^ percentile) responses. Empty circles represent simulation results connected via grey lines, while filled dots represent the corresponding data. Model fit captures percentiles accurately, although it does not account for asymmetries in the Simon effect. (**c**) Same model fit as in (**b**) showing the accuracy in each RT bin, dots mark the proportion of correct responses for each RT bin determined as in (**b**) while the model fit is shown as a dashed line. The congruent condition corresponds to the average of V_L_H_L_ and V_R_H_R_, while the incongruent condition corresponds to the average of V_L_H_R_ and V_R_H_L_. (**d**) Average RT before and after the application of tRNS in our population fitted model. tRNS appears to reduce the average RT in all conditions. The stimulation strength parameter was chosen by examination of (**e**). (**e**) Average *χ* ^2^ across conditions including trials with stimulation, when the model fitted in (**b**) is evaluated for different stimulation strengths. A stimulation strength parameter of 0.022 appears to be the optimal choice. (**f**) When stimulation (*A* = 0.022) is applied to the model fitted to individual subject sham data, an approximately linear dependence emerges between the influence of stimulation and the baseline sham RT (*p* = 0.008). (**g**) Correlation coefficients between each parameter of our model and the sham RT across subjects. The drift term, non-decision time and variability appear to impact the sham RT the most across our model fits (asterisk represents *A* = 0.022). (**h**) The influence of simulated tRNS (*A* = 0.022) on the population fit for different drift parameters (range 0.75 × min to 1.25 × max subject fits). If the real changes in subject RT stem from changes in the drift parameter, then the model predicts that stimulation should be largely ineffective for subjects with low RT, but that at higher RT the influence of stimulation on the change in RT is approximately linear. The inset shows how the drift term (y-axis) varies with sham RT (x-axis consistent with base figure). Note that the simulation was generated using 300 × 10^3^ iterations for improved estimates.

We were interested in whether a simple model of the Simon effect could incorporate tRNS and replicate our findings. Our model (see equations (6–9), depicted in Fig. 4a) when fitted to our sham data (Fig. 4b,c, *I* = 0.279, *x*_*th*_ = 0.404, *t*_*d*_^*base*^ = 0.358, *c*_*td*_ = 0.0615, *b* = 4.93, and ${x}_{0}^{bias}$ = 0.00913) describes the RT in correct and incorrect trials, as well as the percentage of correct trials in each RT bin between congruent and incongruent conditions. Our model captured a key feature of the Simon effect: a weakening of the effect at long RT, including a reversal at the highest percentile we investigated (i.e. the RT distribution has a smaller tail for the incongruent conditions than for the congruent conditions, despite a later mean). The value of the median, as well as the relative increase in RT in the congruent conditions of the mistake distribution RT are also captured. The model may also capture other percentiles of the mistake distribution, but we did not attempt to fit these as variability between the two congruent conditions, and between the two incongruent conditions suggests that our data would not sufficiently constrain the model due to the low number of mistakes. It is interesting to note that one of the main failings of the model is to capture the asymmetry in our data - left vision left hand responses appear to be slightly slower than right vision right hand responses, a robust feature of the Simon effect^35^. Of course, this is not possible in the current implementation as the model is forced to be perfectly symmetrical, however it may be possible to capture asymmetries by allowing small variations in the drift term dependent on condition (as discussed in the following section).

With our fitted model we can address our central question regarding how a DDM based interpretation of the Simon effect reacts to tRNS, which we model simply as additional independent noise (Fig. 4d). We chose a small value of stimulation strength *A*, by scanning this parameter while fixing all other parameters to those previously fitted on the sham data and evaluating the *χ* ^2^ statistic for the entire data set (sham and stimulation). Figure 4e shows how the model fit has a local optimum at a value of 0.022, which we then set as our stimulation strength. Of course, our estimate of the value for *A* is highly dependent on the initial model structure and fit, we therefore use simulations that incorporate the stimulation strength to make qualitative rather than quantitative judgements regarding the influence of stimulation.

It is clear from Fig. 4d, that a general influence of the addition of noise is to reduce RT, although it is unclear whether there is an additional dependence on congruence. Analysis of 100 repeated simulations (each composed of average RT generated from 25 × 10^3^ iterations) demonstrated an expected impact of congruence (slope = 0.0176, 95% Cls [0.0175, 0.0177] assessment by linear model without mixed-effects), stimulation (slope = −0.0020, 95% Cls [−0.0021, −0.0019]), and of the interaction of congruence and stimulation (slope = 0.0005, 95% Cls [0.0004, 0.0007]). However, the influence of the interaction term is an order of magnitude weaker than the direct impact of stimulation, and hence it seems unlikely that it would be detectable under normal experimental conditions.

We were also interested in whether variation in the RT across subjects would impact the ΔRT when ignoring within subject factors, as we showed for our experimental data in Fig. 3a. To investigate this, we fitted our model to individual subjects and then used a stimulation strength *A* = 0.022 as calculated for the pooled data. This is shown in Fig. 4f, where it is clear that simulated subjects with higher RT are associated with a stronger reduction in RT when subjected to tRNS of equal strength. In order to distil the relevant parameter that impacts RT and ΔRT across subjects, we calculated the correlation between RT and each model parameter across subjects. Figure 4g shows that across subjects the drift, non-decision time and variability in non-decision time are strong contributors to the variance in RT. To specifically investigate the influence of tRNS when subject RT is changed due to changes in drift, we used the parameters fitted in the pooled sham model, and varied only the drift term (using a range spanning 0.75 times the minimum fitted drift value across the subjects, to 1.25 times the maximum; Fig. 4h). Given the relatively clear relation between RT and ΔRT induced by changes in drift, and the fact that a change in drift is a strong driver for a change in RT in our subject fits, we suggest that this may be a dominant factor in our experimental observations. We note that the slopes of our simulations in both Fig. 4f,h are not as pronounced as the slope of Fig. 3a, this may be explainable due to an underestimation of the stimulation strength in the model, a plausible explanation if the underlying model structure is not entirely accurate, or simply due to statistical variability in the data or the fitting procedure. We do not show ΔRT dependent on non-decision time (*t*_*d*_^*base*^) or non-decision time variability (*c*_*td*_) since, as might be expected they do not clearly interact with tRNS.

### Within subject response time dependence

If RT is the dominant explanatory variable for ΔRT then it might be expected that when examining within subject changes in RT, congruent conditions should be less influenced by stimulation than incongruent conditions simply because they have lower average RT. This does appear to be the case in our data, as shown in Fig. 3b. However, our model in the form that has been presented so far cannot explain this within subject behaviour. While we can coarsely attribute ΔRT sensitivity to RT, it may be more accurate to consider ΔRT to be driven by changes of many parameters across subjects and conditions. Across subjects, the drift term (*I*) is a strong driver for variability in RT, and it is therefore easy to ignore the contribution of other parameters to ΔRT. However, within a single subject in the model that we have so far proposed, the variation in RT is not driven by changes in the drift term, but rather by the starting decision-variable bias ${x}_{0}^{bias}$, and the slope *b* which differentiate congruent from incongruent trials. It should be noted that the impact of the slope term (1 − *V* · *H*)*b/2* is high in incongruent trials, reducing RT (in order to partially counter the effect of the bias ${x}_{0}^{bias}$) effectively acting similarly to an increased drift term and making the condition slightly less (not more) susceptible to stimulation.

We considered how we might reconcile the behaviour of our model within individual subjects with our results of Fig. 3b. We noted that the main difference between the average RT of our data (Fig. 2b), and the average RT in our model (Fig. 4d) is that the experimental data displays clear asymmetries within congruent, and separately within incongruent conditions, while the model does not. This is a feature of the Simon effect that has been previously studied^35^. We hypothesised that capturing such asymmetries by allowing for variability in the drift term across conditions, either due to response hand or visual hemifield dominance could additionally explain a sensitivity of ΔRT to RT within subjects. For example, upon inclusion of a visual hemifield dominance implemented as a modification of the drift term dependent on condition, it would be expected that the condition with the higher drift (for example V_R_H_R_) would have a shorter RT than its corresponding lower drift condition (V_L_H_L_ in this case), and consequently predict within subject dependence of ΔRT on RT. In other words, the variability between congruent RT and incongruent RT is dominated by ${x}_{0}^{bias}$ and *b* however, in the extended model within congruent and separately within incongruent conditions, the variability is dominated by drift asymmetry.

As discussed in the methods section, we found that when explaining the pooled RT data much of the asymmetry could be explained by using only a single additional parameter to cater for an additional drift component dependent on which visual hemifield the stimulus was presented to. We refer to this model including visual drift asymmetry as the extended model. Upon the assumption that the extended model captures key aspects of our data, not only between subjects but also within subjects, we considered how we might most clearly detect within subject sensitivity of ΔRT to RT. At first sight, it may seem that the most direct approach to determine the presence of this relationship would be to perform individual subject fits, and search for a correlation between RT and ΔRT as we did in Fig. 3b. Interpretation of both our simple model, and extended model warn against this approach however: several parameters with different influences on ΔRT are changing to explain differences in RT between congruent and incongruent conditions. Consequently, a large part of the difference between congruent and incongruent conditions may be insensitive to stimulation. Furthermore, these parameters may not be perfectly recovered in performing individual subject fits which further motivates the need to use a robust feature of our model to compare to our data.

**Figure 5:**
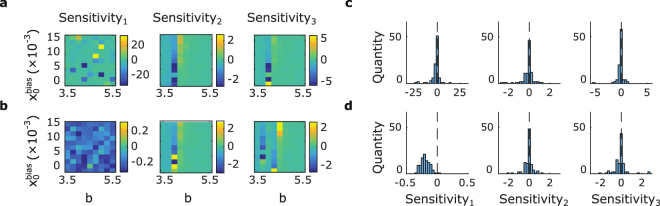
Symmetrical model and extended model predictions for different methods of calculating RT sensitivity. (**a**) Symmetrical model predictions for different *b* and ${x}_{0}^{bias}$ values for different sensitivities. From left to right, Sensitivity_1_ to Sensitivity_3_ as defined in the methods section. With the exception of *b* and ${x}_{0}^{bias}$, all parameters were fixed to the values used for Fig. 4b (pooled data presented for illustration of the rationale behind the sensitivity calculations). Parameters ranges for *b* and ${x}_{0}^{bias}$ were chosen based on individual subject fits, ${x}_{0}^{bias}$ min: 0.000, max: 0.018, mean: 0.009; *b* min: 4.011, max: 5.21, mean: 4.79. (**b**) Same as (**a**) but for extended model with *g* = 0.022, determined as the average from individual subject fits and *I*_*A*_ = *I* from the symmetrical model. Note that for Sensitivity_1_, values are predicted to be consistently negative, while this is not the case in any other condition. (**c**) Histogram representation of (**a**). Sensitivity_1_ median: −0.768 (note that the range of values is large here as between condition RT are very similar, resulting in a denominator near zero); Sensitivity_2_ median: −0.020; Sensitivity_3_ median: −0.016. (**d**) Histogram representation of (**b**). Sensitivity_1_ median: −0.200; Sensitivity_2_ median:−0.015; Sensitivity_3_ median: −0.016. Note that for clarity in these simulations, a stimulation strength of A = 0.044 was used, and colours in (**a** and **b**), as well as x-axis in (**c** and **d**) were clipped.

To this end, we investigated how the simple model behaviour compares to the extended (drift-asymmetry) model with respect to different methods for calculating ΔRT sensitivity. Each plot in Fig. 5a shows ΔRT sensitivity for model simulations with all parameters kept the same as in Fig. 4b, apart from ${x}_{0}^{bias}$ and *b* which are varied systematically. In the leftmost sub-figure ΔRT sensitivity (see methods) is calculated as the ΔRT difference, divided by RT difference within the congruent condition, and averaged with the same calculation repeated within the incongruent condition (Sensitivity_1_). In the central figure it is calculated using pairs across congruent and incongruent conditions, but with stimuli presented to the same visual hemifield (Sensitivity_2_). Finally, in the rightmost figure, pairs are also taken to be across congruent and incongruent conditions but selected for the same response hand (Sensitivity_3_). The figure shows that under the simple model structure, systematic predictions of ΔRT are highly dependent on the specific model parameters (see Fig. 5c). In Fig. 5b we introduce visual drift asymmetry (maintaining all other parameters fixed as in Fig. 5a). We can see that Sensitivity_2_ and Sensitivity_3_ still generate highly parameter dependent values. However, when taking the average of within congruent and within incongruent pairs (Sensitivity_1_), we find that the model consistently predicts values less than zero (see Fig. 5d). In other words, we would predict that the influence of stimulation should be most consistent when examining the sensitivity of ΔRT with respect to changes in RT within congruent, and separately within incongruent conditions (Sensitivity_1_). This is not the case for Sensitivity_2_ and Sensitivity_3_, since they are calculated from RT that are partially influenced by drift-asymmetry, but dominated by ${x}_{0}^{bias}$and *b* which vary across subjects. On the other hand Sensitivity_1_ is predominantly sensitive to drift-asymmetry, and so generates a stable consistently negative value across subjects which is a signature that we can search for in our data. We decided to re-examine our data from Fig. 3b in this light by calculating the various ΔRT sensitivity measures for each subject. This yields a clear negative Sensitivity_1_ (median = −0.76, Wilcoxon signed-rank test, *p* = 0.007, n = 22, z = −2.678). Sensitivity_2_ also appears to be negative (median = −1.01, *p* = 0.039, z = −2.062), although we note that this effect is not as strong as for Sensitivity_1_. Finally, Sensitivity_3_ is not detectably asymmetrical around zero (median = 0.46, *p* = 0.81, z = 0.244).

When repeating the same test using the extended model fitted to individual subjects (A = 0.022, see Fig. 4e), we find Sensitivity_1_ to be negative (median = −0.05, Wilcoxon signed-rank test, *p* = 0.013, n = 22, z = −2.484) as expected, we also find Sensitivity_3_ to be negative (median = −0.02, *p* = 0.006, z = −2.743), but not Sensitivity_2_ (median = −0.02, *p* = 0.131, z = −1.510). As discussed in the previous section, A = 0.022 may be an underestimate of stimulation strength, so we also investigated the behaviour of the model when the simulated stimulation strength was doubled (A = 0.044). In this case, we once again found Sensitivity_1_ to be clearly negative (median = −0.17, *p* = 0.0006, z = −3.425) while Sensitivity_2_ (median = 0.00, *p* = 0.223, z = −1.218) and Sensitivity_3_ (median = −0.01, *p* = 0.249, z = −1.153) did not appear to be detectably asymmetrical around zero. We note that within reasonable bounds, the exact choice of stimulation strength does not impact our main model prediction of a negative Sensitivity_1_ across subjects, which is a robust feature matched in our behavioural data. On the other hand, the fact that Sensitivity_2_ and Sensitivity_3_ are inconsistent between model and data is not unexpected since their RT differences are dominated by ${x}_{0}^{bias}$and *b*, making their predictions highly dependent on individual subjects and individual subject fits as demonstrated in Fig. 5.

### Model selection

In order to provide a statistically rigorous assessment of the different models, we evaluated every combination of progressively more complex model in going from the base model to our extended model (as defined in the Methods section). Every model candidate was associated with a *χ* ^2^ statistic, calculated by performing an 8-fold cross-validated fit, and then averaging for each subject. When computed using cross-validation, the *χ* ^2^ quantifies the model prediction error which can be directly minimized for model selection without the necessity of any additional penalty for model complexity (i.e. number of model parameters)^36^.

**Figure 6:**
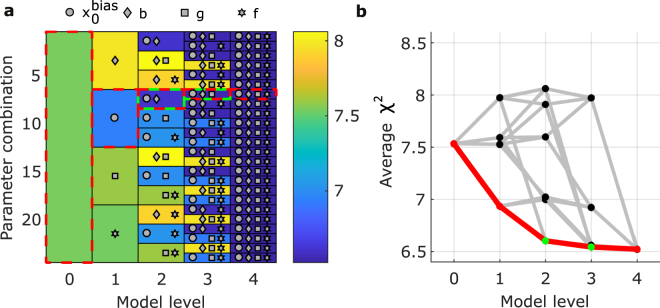
Prediction error of models of different complexity. (**a**) Cross-validated *χ* ^2^ statistic (average across subjects) for base model (level 0), and all proposed model extensions (levels 1–4, corresponding to 1–4 additional parameters). Dashed-red lines indicate the best model at each level. Underlying green outline represents the models used throughout this manuscript. Symbols correspond to additional parameters used in each model (see legend). (**b**) Same *χ* ^2^ statistic as in (**a**), with black nodes representing every model configuration. Red and green colours as in (**a**).

Figure 6a shows how the *χ* ^2^ statistic changes as model parameters are introduced from left to right. For example, the dashed red line shows the progression for the models that we propose and test throughout this manuscript. We start with 0 additional parameters and reduce our *χ* ^2^ the most by adding the ${x}_{0}^{bias}$parameter, followed by the *b* parameter (our simple Simon effect model), followed by the *g* parameter (our extended Simon effect model). The black line in figure Fig. 6b, is equivalent to this progression with all other parameter progressions shown in grey.

Performing Wilcoxon signed-rank tests on the cross-validated subject *χ* ^2^ values, we can see that the addition of the ${x}_{0}^{bias}$ parameter improves the model substantially (chosen level 1 vs level 0, *p* = 0.022, *z* = 2.29, *n* = 22), the further addition of *b* as the second parameter further improves the model (chosen level 2 vs level 1, *p* = 0.001, *z* = 3.30). While the *χ* ^2^ is lower for our chosen level 3 model, than level 2 this difference is not high enough to reach statistical significance (chosen level 3 vs level 2, *p* = 0.661, *z* = 0.44). The level 4 model which captures both visual and response hand asymmetries yields only a slight, statistically not significant reduction in the *χ* ^2^ statistic over our chosen level 3 model (level 4 vs level 3, *p* = 0.548, *z* = 0.60). Model complexity is implicitly penalised in our method, so a reduction in *χ* ^2^ when moving from level 2 to level 4 is not guaranteed. Our aim in moving from a level 2 model to a level 3 model was to capture individual subject asymmetry as we found it in our data (Fig. 3b) and as it has been reported previously^35^. The observed reduction in prediction error supports using the level 3 model, although statistical significance cannot be established with the experimental data of this study. Despite the lack of rigorous statistical support, we consider the choice of the level 3 model justified given its explanatory power regarding the well-established effect of individual subject asymmetry^35^. We note that among level 3 models there is a second level 3 model (using response hand instead of visual hemifield asymmetry) which yielded a very similar prediction error as the chosen level 3 model (Fig. 6). Which of these two level 3 models is indeed superior remains to be resolved in future studies. Likewise we consider whether a level 4 model would be a superior choice over a level 3 model to be work for future studies. Our choice of using a level 3 model over the level 4 model was on the grounds that the level 4 model introduces additional complexity without explaining a new feature of our data. We would like to note however, that the choice of the level 4 model over our level 3 model is unlikely to be critical for our interpretation of tRNS. This is because the additional response hand asymmetry parameter acts in a similar way to the already present visual-hemifield asymmetry to change the evidence accumulation rate across conditions and therefore induce baseline RT dependent changes in RT.

### Conscious and unconscious detection of tRNS

We asked each of our subjects to report on any sensation they felt after each session within an experiment. Out of our 24 subjects, four claimed to notice an unusual sensation in one, or two sessions (‘tingling’, one subject, one session; ‘pressure’, two subjects, two sessions for both; ‘slight burning sensation’, one subject, one session). The sensation of ‘pressure’, is likely to come directly from the electrode montage and be unrelated to the stimulation itself, although the tingling and light burning sensation reported are likely to be in response to the stimulation. Since tRNS clearly went consciously unnoticed by most subjects, in the vast majority of sessions, we do not consider direct conscious sensation to be a likely explanation for our experimental results. It is also not clear why conscious detection should decrease RT only when RT is already long. The lack of conscious sensation in our experiment is consistent with Terney *et al*.^10^ who claim that in their tRNS experiment with the same amplitude, 78 out of 80 subjects were not aware of stimulation.

Although we do not believe that conscious detection plays a significant role in our experiment, it may be that some form of unconscious process (for example, modulation of arousal level by tRNS) is an alternative to a direct influence of tRNS on cortical neurons. While this cannot be ruled out we consider it a less plausible explanation for our results than one involving changes of decision-variables given the fact that we found the influence of tRNS in our paradigm to be dependent on the baseline RT.

## Discussion

We initially hypothesised that tRNS applied across the hemispheres might predominantly disrupt conflict resolution in the Simon task. More specifically we hypothesised that interhemispheric processing is important for conflict processing, and that by applying tRNS across the two hemispheres we might disrupt this processing. Our experiment replicated the typical Simon effect behaviour, however we did not observe a specific influence of tRNS on changes in RT dependent on whether the task condition was congruent or incongruent. Instead, we observed a more general influence of tRNS, which acted to reduce RT.

Interestingly, we also observed a dependence of the influence of stimulation on the baseline RT both between subjects and across conditions in individual subjects. In light of recent work linking the Simon effect to mechanisms similar to those described by models of perceptual decision making^37^, and additional work linking mechanistic interpretations of the influence of tCS to decision making models^20^ we decided to recast our interpretation in terms of a conflict DDM. We found that the DDM based models that we developed can provide a mechanistic interpretation of a reduction of RT due to the addition of noise to the system, and also display changes in RT that depend on baseline RT. The general concept that tRNS may influence RT dependent on the baseline RT, is also supported by explaining the Simon task in terms of a DDM.

A potential explanation for a general mechanism by which tRNS could interact with cortex is by stochastic resonance. This concept has been used to modify tactile sensation in monkeys via aperiodic ICMS^38^, and was used in recent work^12^ to compare visual noise to tRNS, demonstrating an initial increase, followed by a decrease in accuracy in a visual perception task as noise of either modality is increased. While stochastic resonance is an appealing explanation for changes in accuracy, it is a general property displayed by some non-linear systems, and does not incorporate mechanisms of decision making directly, rather stochastic resonance is a property that some decision making mechanisms may exhibit. Here, we are interested in explaining changes in RT so we avoid making direct links to stochastic resonance, and explain our results directly in terms of a DDM based model. While an increase in noise in a DDM should result in a transition along a speed-accuracy curve we chose to focus on RT, despite some indication that accuracies in our paradigm were modified. This is because our subjects were able to consistently perform near 100% accuracy which means that increases, and more subtly, decreases in performance might be undetectable. To see why reductions in accuracy might be undetectable, consider the example that in a DDM operating at 100% accuracy a sufficiently high drift can immunise the choice from an increase in noise. Given this, and the narrow range of accuracies that our subjects displayed, we are cautious to interpret our detected change in accuracy in a single condition with analysis dependent on non-linear models.

In our model, the addition of tRNS, which we incorporate simply as uncorrelated noise acts to reduce the average RT. Essentially this occurs because the addition of noise enhances the asymmetry in the RT distribution which results in a shift of the mean to earlier times. For the basic DDM, it is known that the mean of the RT distribution can be written as^39^: ${\mu }_{RT}\sim \frac{{x}_{th}}{I}tanh(\frac{I\times {x}_{th}}{{c}^{2}})$. It is then clear that as the noise *c* is increased, the average RT *μ*_*RT*_ decreases. Other studies have previously attempted to investigate mechanisms of perceptual decision making, with tDCS or tACS. For example, Bonaiuto *et al*.^37^ model the change in RT due to tDCS during a two-alternative forced choice random dot motion task using a biophysically plausible network model^40^. Their model is based on the model proposed by Wang^40^ represents a decision variable in terms of firing rates of two competing populations with the dominant source of noise in the system being generated from large populations of external neurons. We hypothesise that our conflict DDM could have a similar interpretation at the level of individual neurons and populations of neurons. tRNS would then act to enhance noise in the two populations (and potentially in the external population) by small changes to membrane potentials over very large number of neurons. At the level of our conflict DDM, we speculate that this would manifest as an additional noise term. We believe that our study adds support for the concept that perceptual decision-making models incorporating threshold crossings and evidence accumulation can be probed on-line using transcranial current stimulation techniques. Our primary aim was to explain the influence of tRNS in terms of evidence accumulator models, and not to develop a new model for the Simon effect. Despite that, we believe that the model we have outlined warrants further investigation as a contender for the Simon effect due to its high explanatory power (including the reversal of the Simon effect at high RT, not captured by the DMC^20^), and relative simplicity. It remains to be seen whether like the DMC, our model can also capture the RT distributions in the Eriksen flanker task, or indeed whether it generalises to different variations of the Simon task.

The average left visual hemifield RT change due to stimulation appears to be larger than the average right visual hemifield RT when examining Fig. 2b. The explanation for this may be predominantly due to the weak effects of stimulation, and large experimental noise, combined with the fact that the average left visual hemifield RT are longer than the average right visual hemifield RT. However, we consider the possibility that the influence of stimulation as well as being dependent on RT, is also dependent on the visual hemifield to which a stimulus is presented. This is because the Simon effect itself has been previously shown to display asymmetries which broadly agree with what is shown in Fig. 2b, where generally the left stimuli and left response Simon effect are smaller than the converse^35^. It is therefore possible that natural asymmetries in the processing of visual stimuli under conflict partially explain the different RT that we observe based on visual field dependence. These different RT may then modify the influence of tRNS via the mechanism proposed in our model, however the stimulation itself may also act on asymmetrical processing leading to a mixture of these two effects. In this light, an asymmetrical influence of tRNS on behaviour would not be surprising despite our symmetrical application, although we consider this to be a secondary effect to the general influence of the baseline RT.

The positioning of the stimulation electrodes at FT7 and FT8 in the EEG International 10–20 system, was chosen to interfere with information transfer via corpus-callosum which, if successful may have caused a change in RT predominantly in the incongruent trials. Aside from our initial consideration that tCS might be relatively effective at targeting across the hemispheres due to long axonal projections, the reason for this hypothesis was that incongruent trials require additional inter-hemispheric communication, most likely via corpus-callosum, for example, left visual information is initially processed in the right hemisphere, so if the required response is for a right finger movement, information must additionally cross to the left hemisphere. The idea that there may be an additional delay in inter-hemispheric transfer was demonstrated in the Poffenberger paradigm^41,42^. Here, the authors demonstrated that when healthy subjects are asked to react to a stimulus with the same hand as the side of the visual hemifield in which it appears (congruent condition), their RT are faster than when their reaction must occur on the opposite side (incongruent condition). This result was interpreted as “Splitting the normal brain with reaction time”, the title of work by Filbey *et al*.^43^. This interpretation with its background in split brain research simply suggested that information in the crossed condition must travel via the corpus-callosum, consequently increasing RT. However, the reasons for the delay in the incongruent conditions in the Simon effect task are likely to be different at least in part, as the transmission delay caused by corpus-callosum is much smaller than the changes visible between conditions in the Simon effect task. We consequently consider that tRNS is having a more general influence on decision making circuits. If we are indeed correct regarding our targeting of decision making circuits, then a likely candidate is left dorsolateral prefrontal cortex (DLPFC), which would be covered by our large sponge electrodes, and has been previously implicated as an important region for decision making, which is independent of response modality^44^, and has been previously targeted in perceptual decision making tasks with tDCS^37^.

In our experiment we used broad-spectrum tRNS. Other tRNS studies have been interested in differentiating between high-frequency noise (100–640 Hz) and low frequency noise^10,11^ (up to 100 Hz). For example, it has been shown^10^ that motor excitability after application of high-frequency tRNS, as well as broad-spectrum tRNS (high and low frequency) was enhanced, but not for low-frequency tRNS. Fertonani *et al*.^11^ showed a significant improvement in task performance in an orientation discrimination task when using high-frequency tRNS, and not for low-frequency tRNS, although a direct significant difference in effect strength between the two tRNS types could not be determined. We note that while our model generates qualitatively the same results for all tRNS modalities, it predicts stronger influence of stimulation for low-frequency tRNS, than for high-frequency tRNS due to the low pass filter quality of evidence accumulators. One explanation which may reconcile these various results, is that tRNS exhibits an instantaneous effect caused by direct changes in membrane potential of neurons, consistent with our data and model, as well as additionally having a longer term effect particularly sensitive to high-frequency noise^11^.

We have shown that tRNS can reduce RT in a Simon effect task, particularly when baseline RT are long. We believe that the interaction of noise with models of evidence accumulation that give rise to different baseline responses can explain our results most parsimoniously and have demonstrated this in a novel model of the Simon effect. We did not find any specific influence on the processing of conflicting evidence. We hypothesise that our experimental result may generalise to other types of task, such as two alternative forced choice tasks in decision making during coherent random dot motion that can similarly be described in terms of drift-diffusion models.

## Electronic supplementary material

Supplementary information

Dataset 1
