# Supplementary material for: Modifying response times in the Simon task with transcranial random noise stimulation
Source: Sci Rep. 2017 Nov 15;7:15633. doi: 10.1038/s41598-017-15604-1 (PMC5688076; doi:10.1038/s41598-017-15604-1)
Supplement: Supplementary file 1 — Supplementary information [file 41598_2017_15604_MOESM1_ESM.pdf]

Modifying response times in the Simon task with transcranial random noise stimulation

James Robert McIntosh[1,2,3] and Carsten Mehring[1,2]

[1]Faculty of Biology, University of Freiburg, Hansastr. 9A, 79104 Freiburg, Germany
[2]Bernstein Center Freiburg, University of Freiburg, Hansastr. 9A, 79104 Freiburg, Germany
[3]Imperial College of Science, Technology and Medicine, Department of Bioengineering, South Kensington, London SW7 2AZ, UK.
Correspondence and requests for materials should be addressed to J.M. (email: j.mcintoshr@gmail.com)

Supplementary data description

Data is packaged as a 'csv' file with the following labelled columns:

'ixTrial': Trial number within a block.
'TrialTime': Trial time within a block.
'TrialLength': Complete length of trial measured in seconds.
'TrialLengthCum': Cumulative trial length measured in seconds.
'Stim': tRNS is on or off (1 for on, 0 for off).
'StimTrig': Whether the trial was used to transition from sham to tRNS.
'Location': Location of cue.
'Colours': Colour of cue.
'ReqResponseKey': Required response key.
'ResponseKey': Actual response key.
'ResponseTime': Response time measured from initial stimulus presentation. Please note that a value of 0, marks a non-response ('ResponseKey = 'x").
'subject': Subject number.
'rightHanded': Whether the subject is left or right handed (1 for RH, 0 for LH).
'SlowResponse': Whether the subject took longer to respond than was allowed by the experimental design (value 1 if slow).
'FastResponse' : Whether the subject responded in less than 0.2s.

Please see the manuscript for a full description of the data and experimental conditions. Note that this is the raw data, and dependent on required analysis it must pre-processed. Steps taken in the manuscript for the analysis of RT are: removal of slow responses, removal of incorrect responses, removal of fast responses (e.g. less than 0.2s), removal of responses from left-handed subjects, removal of responses that occur during a transition from sham to tRNS and vice versa.
